# Visual digital intermediaries and global climate communication: Is climate change still a distant problem on YouTube?

**DOI:** 10.1371/journal.pone.0318338

**Published:** 2025-04-08

**Authors:** Alexandra Segerberg, Matteo Magnani

**Affiliations:** 1 Department of Government, Uppsala University, Uppsala, Sweden,; 2 InfoLab, Department of Information Technology, Uppsala University, Uppsala, Sweden; Uninettuno International Telematic University, ITALY

## Abstract

This article addresses the role of digital intermediaries in visual climate communication, and specifically their contribution to the persistence of a ‘green ghetto’ of traditional communicators and repertoires online. We argue for a comparative sensibility: global platforms convey global issues to global audiences, yet the same platform may distribute conditions of visibility for compelling communication unevenly around the world. The study analyses how a major global visual platform, YouTube (Search), articulates climate change in 232 countries in their official languages. It combines API research, channel coding and computational image analysis to assess the processing and presentation of top-ranked results with respect to their diversity and proximity to local context. The findings show that YouTube Search establishes visibility winners who typically sustain the classic visual repertoire of climate change as a distant problem, and that Global North sources dominate irrespective of region. However, there are notable exceptions to these patterns.

## 1 Introduction

It is notoriously challenging to engage citizens on climate change, an issue that is often perceived as abstract and remote in time and place. Strategic recommendations therefore encourage making climate narratives relevant by bringing them close to people’s experience and spheres of concern [1–4]. The recommendations are well known, but observers still note an ingrained ‘green ghetto’ [[2] ch. 6] in communication on climate change, in which a delimited circle of much the same (Global North) speakers, stories, and styles dominate and reinforce the notion of climate change as a distant problem. The primary problem with the green ghetto from the perspective of citizen engagement is not in the first instance what is said about climate change, but how it is said, by whom, and the relation to the audience.

There have been expectations that digital media would dissolve the green ghetto, by supporting fresh voices from beyond the circle of ‘usual suspects’ who might bring repertoires that resonate with local audiences [5–7]. After all, contemporary complex media ecologies soften the grip of editorial gatekeeping, make it easier to produce custom content, enable citizens to seek out issues they care about in interaction with their peers, and amplify visual content, which is known to engage. Empirically, however, it is not clear whether the green ghetto has lost traction online. The initial expectations did not account for the digital platforms that have such a crucial position in this communication landscape [8]. Moreover, studies of online climate communication have tended to focus on text-centred social media such as Twitter, as opposed to the swath of platforms that primarily centre on visual content (e.g. pictures, memes, and videos) such as YouTube, Instagram, or TikTok [6].

In this paper, we are interested in the role of visual platforms in global climate communication and, more specifically, how they contribute to the persistence (or dissolution) of a green ghetto in public discourse online. We approach search engines and social media as digital intermediaries [9,10] that mediate information flows, attention, and connection between climate communicators and audiences.

We focus on rankings (e.g. ranked presentations of content or contributors) as an area in which platform intermediation is overt. Two aspects of rankings are significant: the content that platforms present at the top and the processes that produce these results. With the former, platforms articulate the scope and character of issues, and audiences easily assume that platform priorities in organising information reflect the issue’s core. With the latter, platforms mediate the processes through which issues, actors, and content become prominent, setting conditions for the broader (in)visibility of fresh voices and local connections. We conceive of digital intermediaries in ranking processes as encompassing agencies on several sides. On the platform side, platforms filter, hierarchise, and display content as ranked results, shaped by its politics, policies, algorithms, and affordances. On other sides, core platform doings embed in broader *ranking assemblages* [12]. These also invoke the (re)action of heterogeneous others such as content producers and user audiences.

We argue that a comparative sensibility is crucial to advance understanding of online climate discourse and the role of digital intermediaries within it. Previous work on climate journalism in traditional media observes that the dearth of research attention to Global South contexts is problematic, not least since the societal challenges of climate change call for transnational interventions that require substantial public support [13–16]. In a parallel argument, we propose that a more differentiated knowledge base is important also in the case of online climate communication. To this end, it is crucial to consider not only the differences between digital intermediaries [17], but also *within* them. Although online climate communication is typically studied in a single context (usually US/English), it is not obvious that the operation of a digital intermediary in one context applies more broadly. Indeed, there is reason to expect that ranking processes on global platforms play out differently across contexts. Both the platform itself and other actors involved in the broader ranking assemblages may do things differently across contexts.

This motivates two sets of questions about digital intermediaries in global climate communication, and their role in perpetuating (or dissolving) a green ghetto in public discourse online:

- How do global digital platforms present climate change to differently situated global publics? More specifically, to what extent are fresh voices and local connection present in top-ranked results?
- Do the conditions of visibility for fresh voices and local connection in top-ranked content distribute evenly across contexts?

We address these questions with empirical focus on a major global visual platform that combines both search and social network affordances: YouTube. Pursuing an approach of quantitative description [18] and large-scale observation [12], the study examines how YouTube Search articulates ‘climate change’ to audiences searching for information on the issue around the world. It combines API research, qualitative channel coding, and computational image analysis to analyse and assess the top-ranked search results returned for ‘climate change’ for 232 countries and 85 official languages with a comparative perspective on the underlying processes and results. In order to bring the digital intermediary into focus, we delimit this study to the specifically visual dimension of the platform and videos (i.e. not considering audio or user comments), and assess the presence of a particular set of classic distancing visual themes: arctic landscapes and polar bears.

The results show that top-ranked results on YouTube Search largely perpetuate a green ghetto, with some modification. ‘Usual suspects’ such as professional media continue to figure prominently, now joined by some fresh voices in the form of user-led and digitally native popular science channels. Still, both old and new climate communicators continue to reference classic distancing arctic themes. The results also show that the conditions for local connection are constrained. This is in particular the case for regions that share a global language. Top results for queries in global languages return much the same videos for geographically disparate API search settings (e.g. France, Canada and Cameroon), and channels from one country dominate (e.g. in the case of French, channels based in France). Closer examination reveals distinct profiles in how major language clusters articulate the scope and character of the issue.

The study draws on research in the distinct fields of visual climate communication and algorithm audit approaches in platform studies. In the following sections, we therefore first outline the relevant debates in these two fields and how they inform our research questions, before discussing the case of YouTube Search as a global visual platform in the context of online climate communication. We then proceed to the research design and analysis.

## 2 Visual climate communication and digital platforms

### 2.1 Towards fresh voices and local connection in visual climate communication?

Visual representation, as found in photos, videos, gifs, and memes, is considered a powerful component in climate change communication. Images engage attention and make information more memorable and credible [19–21]. They shape emotional connection to issues and policy [22,23], and underpin a sense of intimacy and direct connection [24]. What is more, under the right conditions, they can transcend linguistic and national borders [25,26]. This means that visual communication can be key to bringing climate issues close to home.

Conversely, visual content can also bolster the impression that the issues of climate change are abstract and remote in time and place. Ironically, a recurring element in visual climate discourse is the distancing frame [[27] p. 16], which is broadly assumed to be problematic for engaging people with climate change. The distancing frame reinforces the idea that climate issues are geographically, temporally, and psychologically remote from everyday life. Typical examples include the classic visual themes of political or celebrity elites, polar bears, and empty (arctic) landscapes that became prominent and entrenched at the turn of this century in Global North civil society advocacy campaigns [28], stock footage infrastructures [29], legacy popular science media and climate journalism [27,30–33]. The evidence for the underlying psychological theory is mixed, and audience perception studies diverge on exactly when and how climate images are experienced as distancing [34–36]. However, a range of studies suggest that many find the classic repertoires distancing, and some audiences more so than others. There is experimental support for abstract images (e.g. empty landscapes) being more likely to create a perception of climate change as spatially and temporally remote [37]. Moreover, observational studies find that audiences in the Global North interpret the classic distancing visual themes associated with mass media climate reporting as salient but demotivating [38–40]. For some, the iconic visual themes have become worn clichés that associate with cynicism and fatigue [38,40,41]. In addition, while images travel, audiences also interpret the same image differently. For indigenous communities and audiences outside the Global North, the themes can be far removed from both familiar visual repertoires and lived experience [42].

Accordingly, recommendations for compelling visual climate communication echo the general call for communicators to prioritise speakers and content with relevance to the audience at hand. The recommendations for visual content emphasise relatable proximity in actual depictions: the advice is to show ordinary or ‘real’ people in authentic (not staged) situations and scenes with local connection [1,41,43]. More generally, recommendations emphasise the value of deploying fresh visual narratives (not one-size-fits-all clichés), and being sensitive to ways in which visual themes, references, and repertoires may be so culturally loaded that they are not readily relatable outside that context [1,4,25,41]. Testing such principles in the digital sphere, an analysis of 380 images in Twitter ‘top tweets’ confirmed that user engagement in comments, likes and retweets was higher for climate change messages that contain images with real people and include a local connection [3].

In principle, such findings suggest that the digital context may offer an organic path away from the green ghetto of usual suspects and themes. It is easy to assume that the ability to source one’s own visuals would make it easier, and perhaps more likely, to tell stories with local themes [7]. It would follow that, if a variety of communicators use their own sources to tell stories, if images with ordinary people and local connection are popular, and if we factor in how visuals increase the spread of messages [44] and how imitation features in networked visual publicity [45], then online climate discourse would become more varied and usual suspects and timeworn distancing themes less dominating. Yet, this is where digital intermediaries complicate the picture. What climate communicators post is one thing, the visibility afforded them is another.

### 2.2 Diversity and proximity in online rankings

An important part of what platforms do in the world is present issues to audiences. Research in platform studies emphasises the persuasive power inherent in how search engines and social media present actors and content. User audiences stay with default settings [9], and may not consider who or what is not included. They respond to signals of salience and urgency in engagement metrics and trend tickers [46–48]. They pay attention to, and ascribe authority to, posts positioned at the top [49,50]. Rankings — for which platforms filter, hierarchise, and display actors and content — become pivotal in suggesting the shape, scope, and relevance of issues.

Algorithm audit research helps investigate this aspect of what rankings do. Such work systematically probes algorithmic workings in the light of societal implications [51,52], for example with respect to how rankings amplify gender and race bias [53], misinformation [54], memory politics [55], and political information bias during election periods [56].

Two strands in this literature are pertinent for assessing the persistence of a green ghetto in top rankings. The first centres on the (lack of) diversity at the top, which has bearing on the extent to which fresh voices gain prominence. This work suggests issues of composition (which actors) and concentration (their distribution) can be crucial. A number of considerations may play into making results relevant, including freshness, user satisfaction, and introducing fresh content by serendipity (while perhaps balancing with sponsorship). The evidence challenges common assumptions about the constraints on diversity in typical ranking logics [57]. However, studies also present instances in which particular platform ranking practices privilege large or already popular sources while under-representing others [58], concentrate attention to a select number of sources [59], or skew content due to differences in what high- and low-ranked sources present [60].

The second strand examines the relevance of top-ranked content for differently situated audiences in terms of proximity, which has bearing on issues of local connection. Importantly, this work illuminates systematic variations in the ‘localness’ of top-ranked content (beyond personalisation). Platforms roll out features and moderation technologies at different pace across locations or in keeping with local regulation [61]. The same arrangements may also produce distinct results in different contexts. In one study, for example, Google Search returned city information with different localness of sources depending on how developed publishing and scientific industries were in the respective regions [62]. In a different take on local connection, another study found that object recognition systems of the kind that underpin image search performed poorly in recognising household items such as spices from non-Western and low-income communities [63].

These two strands inform our approach to the question of how platforms present climate change to differently situated publics, and specifically the degree to which top-ranked content includes fresh voices and local connection. We specify our first question as follows:

(RQ1) *What characterises top-ranked results for climate change with respect to diversity and proximity on YouTube Search — and does this differ across region/language search contexts?*

### 2.3 The conditions of visibility across contexts

Another important part of what digital intermediaries do hinges on the processes that produce top ranks. If we want to understand the role of digital intermediaries in climate discourse in light of the green ghetto, we need to consider not only whether fresh voices and local connection appear in the top results, but also what the conditions of visibility are for this to occur. Here, the workings of the digital intermediary go beyond ‘the’ platform or ’the’ algorithm to also encompass actors such as the users that post content and the audiences that engage with it.

Research on how algorithms intervene in the world emphasise the socio-technical dimension [64]. A strong case is made for acknowledging the distributed agencies that converge as assemblages in ranking processes [12], what we here refer to as ‘ranking assemblages’. On this view, ranking processes are embedded in, and are an entanglement of, platform politics [65] and use cultures [66]. Intensifying the multi-sided tangle, interaction and cues run in several directions. For example, between algorithms premised on user engagement (among other things), actors that post content and anticipate such curation (among other things), and audiences that discover content to like. Consequently, in specific assemblages, some outcomes become more likely and others less so, which circumscribes the conditions of visibility in that context.

Taking this starting point seriously implies that it makes sense to consider ranking assemblages and their conditions of visibility in a comparative perspective. Given that not only platforms but also other actors imprint ranking assemblages, we can expect differences across contexts in several tangents. On the one hand, as noted, platform-side doings may in fact vary across contexts — or, a platform doing the same thing may integrate locally to different effect. On the other hand, what content contributors and audiences do also plays in. The intuition about varied patterns is supported by emerging research. An exploratory cross-platform study identified distinct visual vernaculars associated with climate change across platforms [17]. Related work (not on climate issues) suggests that there is also reason to expect differences *within the same platform*. In the case of YouTube, a handful of studies show that ranking cultures differ across issues (e.g. have a stable or ‘newsy’ morphology [12]), and take different shape around the same issue across language/region contexts [67,68]. Such suggestive findings motivate the question whether the conditions for dissolving a green ghetto are the same everywhere on a platform such as YouTube. We specify our second question as follows:

(RQ2) *What characterises ranking assemblages underpinning top-ranked results on climate change on YouTube Search — and does this differ across region/language search contexts?*

The multi-sided interactions in ranking assemblages are inherently difficult to pick apart. Nevertheless, we argue that they are still accessible to comparative analysis. In this study, we follow the proposal to address the tangled agencies through API-based large-scale observation [12,67]. This, like most other approaches to algorithm auditing, leverages outside observation to handle the challenges of access, transparency, comprehensibility and randomisation in dealing with algorithms [51]. We recognise that other approaches could be more powerful for focusing particular relations (e.g. agent-based audit to focus user-platform dynamics [69]). However, an advantage of API observation for our purposes is that it makes it possible to systematically probe the multi-sided interactions in how they unfold over time, and thereby lends traction to comparative analysis. Since the API for a platform such as YouTube Search runs as close as possible to a baseline search, the API approach is also useful when the analytical focus is the platform-level process rather than what specific users actually see [12,70].

## 3 Climate change on YouTube search

We focus on YouTube, a video sharing and social media platform that is receiving increasing attention for its pivotal position in global online climate discourse. The U.S.-based platform is a global destination for climate news, science, entertainment, and education content that spans the spectrum from professional to amateur [71,72]. It is localised in over 100 countries and 80 languages [73]. A sizeable audience is in the U.S., which is the most studied. By 2022, however, YouTube was twice as big in India, and had major markets in countries such as Indonesia, Brazil, Russia, and Japan, followed by others such as Mexico and Germany [74]. The platform offers several popular ways to discover, navigate and consume content, including Search, following recommendations for related content via the UpNext listing, or subscribing to particular channels.

Search is a central affordance on the platform. The default setting ranks results by ‘relevance’, displaying thumbnail image, title, views and date uploaded. YouTube prioritises three key considerations in connecting users with content in Search: the *relevance* of keyword match in titles, thumbnails, video description and content; *viewer satisfaction* in aggregate signals of engagement such as watch time, likes, and comments; and *quality*. For quality, the platform surfaces authoritative sources and demotes borderline content, especially for news, politics, science, and medical information. Personal search and watch history contribute to results if a user is logged in [75,76]. As noted, ranking assemblage differences have been observed within and between other issues on YouTube Search [12,68].

YouTube has been accused of being both too lax and too censoring of actors and content (in different contexts) [77]. If global search results on climate change follow the patterns from English/US studies on mainstream climate search terms, we would expect them to be relatively conservative: they would be primarily characterised by ‘usual suspects’ and classic visual themes. Previous research on US/English samples finds that news media channels are prominent, and that they dominate among those showing classic distancing themes such as the ‘disaster impact’ frame [70,71]. We can expect continuities across regions on this point. Climate change is precisely the type of issue for which YouTube elevates authoritative sources such as news media, and research on global climate journalism shows that while there are region-specific trends, there is continuity across countries in common news frames, including the distancing frame [13,15]. Regarding the user-led channels, studies of YouTube Search indicate that they also appear in top results, and that they tend to be more popular [70,78]. However, it is less clear what to expect from them with respect to local connection in content. A qualitative study with focus on ‘gain’ and ‘loss’ frames observed that user-led climate channels tend to re-inscribe legacy media repertoires into their videos [79], but the study on the disaster impact frame did not note user-led channels using this frame in top results [70].

## 4 Method

This study pursues an approach of quantitative description [18] and large-scale observation [12,67] to examine what YouTube Search presents in top rankings and the underpinning processes with respect to climate change. For our first question, about platform presentation, we consider the channel actors and visual content in top ranked results. For the second question, on the underpinning processes, we consider the platform, channel, and audience sides of the ranking assemblages.

### 4.1 Delimitations

As we detail in the following sub-sections, we make several key delimitations in order to focus platform-level processes in this context. Chief among these include the following:

- We analyse only the visual dimension of YouTube and its videos, and do not consider other aspects such as the audiovisual elements and user commentary.
- We look at API results, not at what specific users would see when searching (e.g. based on their search history or other criteria that YouTube might be using but not corresponding to specific API settings).
- We focus on a keyword that has been shown to have a mainstream profile on YouTube search, ‘climate change,’ [71], since we expect green ghetto discourse to characterise mainstream communication. For this reason, in this study we have not used other keywords from the literature, such as ‘climate hacking’ or ‘chemtrails’, better suited to the study of specific aspects within climate change and/or specific groups of producers and audiences. Practically, using a general keyword also simplifies the translation to other languages, which is an important factor given the world-wide scope of our research.
- We examine only one kind of distancing theme (in its arctic rendition), which is selected to give us leverage on the comparative analysis of relevance in terms of freshness and localness.
- We approach the image data as still images, and do not consider their sequence since we are only looking at which videos show this type of content.
- Finally, although we are working with a sizeable dataset, our observations are by design based on small sample sizes, since we focus on the videos that the platform ranks at the very top across contexts. Our results are consistent across data collections performed at different times, which allows us to make observations regarding the presence or absence of specific themes and actors in the search results. However, we do not use our data to support statistically significant statements such as the fact that the proportion of videos showing arctic themes for one country is higher or lower than for another.

### 4.2 Data collection

The data for this study was collected from the YouTube Search API in May 2021. The collection retrieved search results for all countries that can be specified in the API in their main official languages, based on videos published up until December 2020. We decided to use all languages that are listed as official for at least one of the country codes that can be specified in the YouTube API in order to be as complete as possible. We achieved satisfactory coverage of 232 countries and 85 languages, despite not being able to find translations for a few languages (which we document in the data released with the study). The accounts used to retrieve the data are set to not use personalisation.

We used the following API settings:

- We set order by ‘relevance’, the default search setting.
- The search term or query (‘q’) was ‘climate change’ or its equivalent in each respective language.
- Searching for all official languages meant that for some countries we made multiple distinct queries. For example, in the case of Canada, we searched for both Canada/English/‘climate change’ and Canada/French/‘changement climatique’.
- We used the RegionCode parameter to pass country information to the API. This parameter only retrieves videos that can be viewed (i.e. are not blocked) in the specified country.
- We did not use the *location* and *locationRadius* parameters, which constrain the results to channels located in the specified radius, since they do not seem to be used in the default search settings.

The data consists in the ranked results of 276 queries, which cover 232 countries and 85 languages. Our self-imposed limit was 400 results per search. We here only report findings based on those region/language pairs with 400 results. 253 queries returned the full 400 results, covering 219 countries and 62 languages. We focus the analysis particularly on top-20 rankings [49,50].

### 4.3 Data preprocessing: Channel coding and image annotation

To prepare the channel data, we coded all channels with videos in top-20 rankings by actor type, based on channel description. The coding scheme of 17 categories builds on previous work [70,80], to include actors that are conventionally prominent or studied (e.g. legacy media, science, and civil society organisations), traditional actors that have conventionally been less prominent (e.g. large businesses, political parties, public agencies, religious organisations), and actor types created for this context (e.g. user-led channels, online popular science and education actors). The inter-coder reliability between two coders on a random sample of 10% of a larger data set of 1631 channels was Cohen’s kappa .81 (see supplementary material: coding schemes, channel categories). While the agreement was high in general, there was some disagreement on the code Popular science group, which we note here because this category became important in the analysis. This disagreement was analysed and resolved by discussion, as it had to do with miscommunication regarding the focus on non-institutional groups dedicated to popular science communication (as opposed to individuals, public intellectuals, or school classes).

For the visual analysis, we used a method centred on computational image analysis to assess the presence of a distancing visual theme that has been strongly represented in conventional climate communication such as mainstream media reporting. We sought a means of analysing local connection in visual repertoires as part of assessing a potential break in convention and move towards relatable climate communication. Because of the difficulty in achieving robust large-scale comparative coding of ‘local’ themes, we focused solely on the opposite to local, i.e. the presence of a classic distancing theme. We selected the distancing theme in its arctic rendition: polar bears, icebergs, and ice cliffs in water. Building on the literature outlined earlier, we assume that many find the arctic themes not relatable. This, because these classic themes are geographically remote (for most), devoid of people, and specific to a particular visual repertoire tradition. Moreover, the use of the classic arctic themes suggests thematic continuity rather than innovation.

To prepare the visual data for computational image analysis, we segmented the videos into shots (contiguous frames from an individual camera) and selected the central frame from each shot, resulting in about 150 000 images for a set of top-20 results. Following a procedure reported in greater detail in the Supplementary material, to identify polar bears, we classified the images using a combination of pre-trained and custom deep neural networks, all with a single corresponding label. We also added polar bear images that we discovered in the Google Vision API search for ice images, and manually eliminated false positives. For icebergs and cliffs, we used Google Vision API to identify frames with ice (and related labels) and manually coded the resulting data according to a visual code book (see supplementary material: coding schemes, visual themes). The inter-coder reliability between two coders on this material was Krippendorf’s alpha .84. The high reliability score can partly be explained by the decision to only consider unequivocal depictions of the themes. For example, pictures coded as icebergs must show an iceberg in a prominent position, with the ice clearly protruding from the water (the visual codebook and examples of positive and negative images are provided in the Supplementary material). We note that this approach yields a conservative assessment of the presence of the themes: allowing broader definitions of these themes would have led to an even higher amount of content being coded as icebergs and cliffs.

### 4.4 Assessing diversity and proximity in top-ranked results

We draw on the channel coding and image analysis just outlined to assess the platform presentation in terms of diversity and proximity (RQ1). To assess diversity, we analyse both the concentration and composition of channels. We consider concentration of channel types using entropy and classification error as measures to examine whether the platform presents a broad or narrow set of voices in top-ranked results. We analyse the composition of channel types to examine the diversification of voice beyond traditional climate communicators (see supplementary material: measures).

To assess proximity in top-ranked results, we analyse the local connection of contributing actors (i.e. channels) and visual themes in the video content. To analyse localness of channels, we refer to channel metadata about source location. For visual themes, as discussed earlier, we focus on classic arctic distancing themes as preeminent *non*-local motifs. We analyse the proportional prevalence of these specific themes both in top-20 videos and across lower-ranked results.

### 4.5 Examining ranking assemblages

To examine the conditions of visibility in the underlying ranking assemblages across contexts (RQ2), we undertake systematic large-scale observation [12,67] with focus on platform-, user-, and channel-side factors.

As noted earlier, it is difficult to disentangle the various agencies that contribute to ranking assemblages. Nevertheless, we argue that it is possible to systematically probe the multi-sided contributions in a comparative perspective. The systematic analysis pivots on a logic of potential position. In brief, we assume a space of possibilities for how platform, channel, or user (i.e. audience) agency could in principle influence the ranked content, then locate the actual position in the top-ranked results. We focus on the potential of a particular side to contribute to diversifying the ranking in ways relevant to dissolving the green ghetto on a platform such as YouTube. To dissolve the green ghetto, platforms need to surface fresh channels and themes, but it is also the case that channels need to bring fresh content and audiences need to engage with it.

For the platform side, we analyse the extent to which the same query yields different results across time and place, with an eye to the visibility given to particular types of channels and the conventional arctic visual themes. We assume that the platform in principle could actively diversify results for the same query at different times and could return locally relevant results at different locations. For the temporal analysis, we execute the same queries at different times, from a few seconds to three months apart, then compute the overlap between top-ranked results as the number of common top-20 videos divided by 20 (see supplementary material: diversity, time). We only requested videos published until December 2020 (several months prior to the data collection) in order to avoid contaminating the results with recent videos not available to previous searches. To assess localness in the platform context, we take all available region/language pairs query results, and examine location indirectly, through language (expecting there to be a correlation between channels and videos with metadata from regions where that language is spoken), and directly, by analysing the effect of the *regionCode* parameter, which returns only videos that can be viewed in the specified country (videos can be blocked in a specific region either by the channel or by the platform).

For the channel side, we assess if diversification of (channel) voice brings diversification of visual content by examining whether all channel types in the same context have the same probability of showing the arctic visual themes. Similarly, to probe the role of user audiences, we consider diversification in channel preferences. We assume that if audiences strongly prefer a channel, this would favour its visibility in the top (even if channels with little engagement also appear). We take engagement in views, comments and likes as proxy for the broader set of user popularity signals that platform ranking algorithms may draw on. The YouTube data only provides global metrics for each video, but we can still compare relative preferences across contexts.

## 5 Findings

**Table 1:** Composition and content of the combined search results from all country-language pairs, considering the top-20 results (videos) for each pair. #res: number of search results. %res: fraction of search results. #cha: number of distinct channels. #vid: number of distinct videos. amp: fraction of search results divided by fraction of videos minus 1. #vie: median number of views per video. #com: median number of comments per video. #lik: median number of likes per video. %PB: fraction of videos showing polar bears. %Ice: fraction of videos showing icebergs or ice cliffs in water. %PBI: fraction of videos showing polar bears, icebergs or ice cliffs in water. Only categories with at least 2% search results.

Category	#res	%res	#cha	#vid	amp	#vie	#com	#lik	%PB	%Ice	%PBI
All Categories	5060	1.00	1067	1369	0.00	2785	34	2	0.08	0.18	0.20
Prof. media org.	1932	0.38	310	436	0.20	3402	39	3	0.08	0.20	0.22
User-led channel	965	0.19	183	199	0.31	13778	192	32	0.13	0.27	0.29
Pop. sci. groups	424	0.08	43	55	1.09	28369	542	63	0.15	0.31	0.31
No description	397	0.08	199	222	-0.52	706	10	0	0.07	0.13	0.14
(Inter)gov. org.	317	0.06	80	131	-0.35	1253	10	0	0.02	0.13	0.14
Civil society	245	0.05	74	93	-0.29	841	10	0	0.06	0.11	0.13
Online education	235	0.05	41	48	0.32	8516	117	8	0.17	0.12	0.21
Business	119	0.02	32	35	-0.08	3595	23	3	0.09	0.14	0.17
Science org.	110	0.02	44	52	-0.43	1240	10	0	0.06	0.13	0.15

### 5.1 Diversity and proximity in top-ranked results

We first consider how YouTube articulates climate change to global audiences: what characterises the top-ranked results on YouTube Search with respect to diversity and proximity, and does it differ across contexts (RQ1)? The findings show that on YouTube, the issue is still typically a distant problem delivered by a constrained circle. In general, the issue is defined by a delimited set of channel types, who do in fact expand beyond the usual suspects but still sustain a traditional distancing visual repertoire. This said, it depends on where you look. Closer examination reveals that language groups articulate the scope and character of the issue with notably distinct profiles.

The aggregate analysis focusing on the top-20 results suggests that YouTube supports ‘visibility winners’ [67] on climate change. A concentrated set of channel types dominate top results, and their videos are consistently more likely to top search results across different regions. As Table 1 shows, media organisations are the most prevalent, with a higher fraction of videos in the top results. Their videos are highly amplified — the API returns the same video in several regions’ rankings — but not the most so.

Actors fresh to conventional climate communication are also highly amplified, and audience engagement with these channels is higher than for professional media channels. User-led channels are relatively prevalent at the top, highly amplified, and (often) highly engaged with. Videos posted by digitally native popular science groups and online education actors are the most amplified, although with limited prevalence in top results. For example, the same German popular science channel appears across several global regions. These popular science and education channel types are also the most engaged with. By contrast, other traditional climate communicators have little visibility in the aggregate results. Authoritative channels such as (inter-)governmental and science organisations, and potential stakeholders, such as political actors, civil society, and religious organisations, are neither prevalent, amplified, nor engaged with.

Nevertheless, the iconic themes of the distancing frame continue to be part of the climate narrative. One fifth of the search results show classic arctic themes (and recall that we are using a conservative coding for these themes). While fresh actors from beyond the conventional climate communication circle do appear in the top ranked content, they do not noticeably reduce the prevalence of the arctic theme. If anything, fresh climate communicators support its persistence. Table 1 shows the extent to which videos in the respective channel categories include the themes. Professional media maintain arctic content, and online education actors match this. Yet, the other newly prominent actors, user-led channels and popular science groups, surpass these levels. By contrast, traditional communicators such as science organisations, civil society, and (inter-)governmental organisations show little of these themes, but as just noted, are also not particularly visible themselves.

Beyond the aggregate, there is significant variation across contexts. Language is the main differentiating factor, as opposed to geographical location on the globe or categorisations such as Global North/South. Recall that the data is collected through the Search API with no personalisation: individual YouTube users may see different results, which is not what we assess in this study. The top results evince different profiles, and channel types behave differently across contexts. We here therefore present the results according to language groups.

**Table 2:** Composition and content of search results for selected languages. Channel concentration is computed using the concentration measure based on entropy (supplementary material: measures), with higher values indicating higher concentration of the results towards a smaller set of categories (and thus lower diversity).

Arabic									
Channel concentration: .41. Content: .08 (PB) .30 (PBI).									
Lang	Category	#vid	#cha	#vie	#com	#lik	%PB	%Ice	%PBI
ar	Prof. media org.	27	15	3367	41	4	0.07	0.22	0.26
ar	Pop. sci. groups	5	4	443622	7840	804	0.00	0.40	0.40
ar	User-led ch.	3	3	4658	114	22	0.00	0.00	0.00
ar	(Inter)gov. org.	2	2	5916	58	2	0.50	0.00	0.50
ar	Dig. media prod.	1	1	204555	3239	109	0.00	1.00	1.00
ar	Online education	1	1	17544	664	39	0.00	0.00	0.00
German									
Channel concentration: .39. Content: .15 (PB) .22 (PBI).									
Lang	Category	#vid	#cha	#vie	#com	#lik	%PB	%Ice	%PBI
de	Prof. media org.	20	15	189056	3667	1206	0.15	0.20	0.20
de	No description	2	2	221063	5089	2768	0.00	0.00	0.00
de	User-led ch.	2	2	15856	279	132	0.00	0.50	0.50
de	Civil society	1	1	5413	210	5	1.00	1.00	1.00
de	Dig. media prod.	1	1	285431	4017	916	0.00	0.00	0.00
de	Music	1	1	418060	712	26	1.00	0.00	1.00
de	Online education	1	1	226329	2553	986	0.00	0.00	0.00
de	Pop. sci. groups	1	1	295842	9558	2457	1.00	1.00	1.00
de	Religious org.	1	1	285420	2273	30	0.00	0.00	0.00
de	Science org.	1	1	5222	174	38	0.00	0.00	0.00
English									
Channel concentration: .34. Content: .10 (PB) .29 (PBI).									
Lang	Category	#vid	#cha	#vie	#com	#lik	%PB	%Ice	%PBI
en	Prof. media org.	43	32	47974	785	329	0.05	0.30	0.30
en	User-led ch.	25	24	89116	4689	540	0.08	0.32	0.32
en	Extr. weather ch.	5	2	42125	686	131	0.00	0.00	0.00
en	Pop. sci. groups	5	3	4574151	243961	22929	0.00	0.20	0.20
en	(Inter)gov. org.	3	2	4000	119	9	0.00	0.00	0.00
en	Business	2	2	122354	1238	34	1.00	1.00	1.00
en	No description	2	2	923957	14904	2843	0.00	0.00	0.00
en	Online education	2	2	299392	3138	98	0.50	0.50	0.50
en	Talk platform	2	2	685784	14126	2388	0.00	0.00	0.00
en	Civil society	1	1	806	21	0	0.00	0.00	0.00
en	Science org.	1	1	21123	211	119	0.00	0.00	0.00
French									
Channel concentration: .13. Content: .14 (PB) .38 (PBI).									
Lang	Category	#vid	#cha	#vie	#com	#lik	%PB	%Ice	%PBI
fr	Prof. media org.	12	10	40314	452	44	0.00	0.33	0.33
fr	No description	6	5	6420	124	18	0.50	0.50	0.67
fr	User-led ch.	5	5	56007	2683	475	0.00	0.00	0.00
fr	Pop. sci. groups	4	3	143014	1041	12	0.00	0.25	0.25
fr	(Inter)gov. org.	3	3	26626	3	0	0.00	0.33	0.33
fr	Civil society	3	2	38318	393	19	0.33	0.33	0.33
fr	Politician, party, …	2	2	14362	2117	300	0.00	0.00	0.00
fr	ch. discontinued	1	1	11793	182	130	1.00	1.00	1.00
fr	Science org.	1	1	19748	238	160	0.00	1.00	1.00
fr	Talk platform	1	1	13214	241	113	0.00	0.00	0.00
Japanese									
Channel concentration: .05. Content: .15 (PB) .25 (PBI).									
Lang	Category	#vid	#cha	#vie	#com	#lik	%PB	%Ice	%PBI
ja	User-led ch.	5	4	22099	783	58	0.00	0.00	0.00
ja	Prof. media org.	4	4	15662	194	42	0.25	0.75	0.75
ja	Business	3	3	9485	103	4	0.00	0.00	0.00
ja	Science org.	3	1	43567	534	0	0.33	0.00	0.33
ja	(Inter)gov. org.	2	2	8007	62	6	0.00	0.00	0.00
ja	Pop. sci. groups	2	2	73106	2133	227	0.50	0.50	0.50
ja	Civil society	1	1	1224	118	3	0.00	0.00	0.00
Portuguese									
Channel concentration: .11. Content: .00 (PB) .02 (PBI).									
Lang	Category	#vid	#cha	#vie	#com	#lik	%PB	%Ice	%PBI
pt	(Inter)gov. org.	9	9	2289	133	1	0.00	0.00	0.00
pt	Civil society	7	6	841	22	3	0.00	0.00	0.00
pt	No description	4	4	1262	14	0	0.00	0.25	0.25
pt	Pop. sci. groups	4	3	548	114	0	0.00	0.00	0.00
pt	Online education	3	3	37395	1315	0	0.00	0.00	0.00
pt	User-led ch.	3	3	13778	3401	135	0.00	0.00	0.00
pt	Prof. media org.	1	1	2829	60	0	0.00	0.00	0.00
pt	Religious org.	1	1	11263	301	34	0.00	0.00	0.00
pt	Talk platform	1	1	1360	87	0	0.00	0.00	0.00

The English-language queries, as the largest cluster, follow the aggregate profile. As Table 2 shows, the top-ranked results concentrate to a small number of channels. ‘Climate change’ is primarily defined by professional media and user-led channels, and to some extent also popular science groups. The top-20 channels tend not to be local: channels from only 14 countries are represented in the 79 English-language searches, and we note a clear dominance from the US and UK. Almost one third of the top-ranked videos include arctic distancing themes.

Beyond English, the results in the language groups span a spectrum of profile types. Top results in French and Japanese distribute relatively evenly across channel types. At the other extreme, Arabic and German render climate change as an issue highly defined by one type of channel. In these examples, the channel type in focus is professional media, but this is not always the case in concentrated profiles. For example, in other (smaller) languages with concentrated profiles, (e.g. Turkmen, Zulu) user-led channels dominate (see supplementary material: top-ranked results).

Among the globally spoken languages, sources based in Europe often dominate. In the French cluster, the top-ranked videos are almost all posted by French (and some Belgian and Swiss) channels. This means that top-ranked results, which have a relatively high prevalence of arctic content (38%), are not local to other regions that get these results, such as, Canada, Cameroon, and Haiti. In the Spanish language cluster, which has levels of arctic content more akin to English (27%), channel sources spread between Spain and Latin America. Nevertheless, Spain contributes the most channels. This is twice that of Mexico, despite Mexico being the biggest YouTube market in the cluster.

By contrast, large markets with their own language have a larger portion of local channels at the top. Japanese, German, Hindi and Indonesian queries (almost) exclusively present sources with local connection. Russia is an exception, with only half the videos stemming from Russian channels and the others from a range of eight countries. There is also a different pattern for Hindi and India/English: the former strongly features online education channels and channels with local connection, the latter aligns with the English cluster pattern of skewing towards media, user-led, and popular science channels, and only three are local. Still, in all of these languages — large markets but small YouTube languages — the arctic themes persist in up to one third of the videos (from Indonesian at 20%, German 22%, Japanese and Hindi at 25%, to Russian at 29%). For the Arabic cluster, in which most top channels are regional news media or localised versions of global broadcasters such as BBC, Deutsche Welle, and France 24, arctic themed content sits at 30%.

The real standout is Portuguese. This is the only global language cluster in which reference to climate change as a distant problem via the arctic theme is virtually absent. Only one video, an animated infomercial posted by a No Description channel, depicts an arctic theme. This is also the only cluster dominated by channels local to a single Global South country, Brazil. The issue is articulated through a highly distributed set of channel types that inverts the otherwise typical profile: (inter)governmental and civil society channels are prominent, explicitly user-led channels less so, and professional media presence is limited to one single video.

### 5.2 Conditions of visibility: Ranking assemblages across contexts

We now dig into the conditions of visibility underpinning the top-ranked results and how they contribute to sustaining or dissolving the green ghetto: What characterises ranking assemblages underpinning the top-ranked results for ‘climate change’ on YouTube Search, and does this differ across region/language search contexts (RQ2)? Our findings suggest that the ranking assemblages, and the conditions of visibility that they entail, are fairly constant. On this mainstream climate keyword, ranking assemblages operate in largely similar ways across contexts, and this in turn translates into different prospects for fresh and locally relevant climate communication in particular contexts. We probe the platform, channel and audience sides of the ranking assemblage, and find that agencies on the respective sides contribute.

**Fig 1:**
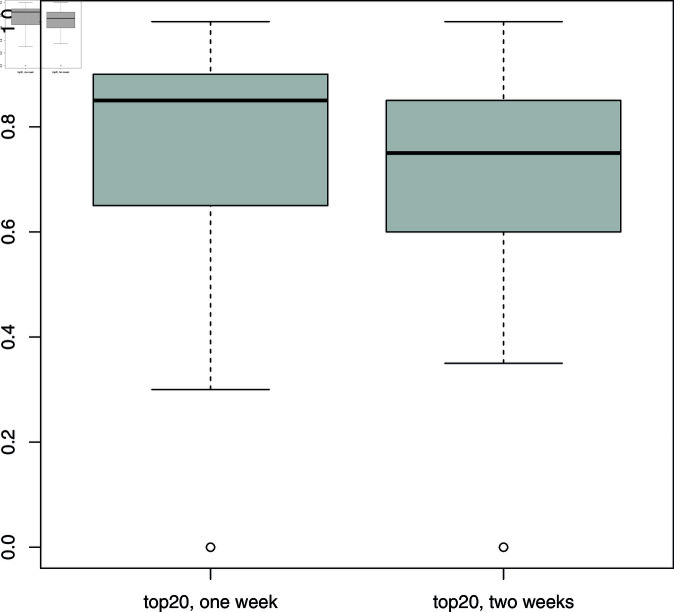
Platform’s temporal diversification. Value 1 corresponds to the case where results of the same country-language query obtained at different times are identical. Value 0 corresponds to query results with no common videos. The plot shows the distribution of temporal diversification over country-language queries.

To start with the platform side, we do not observe the platform actively diversifying results or giving visibility to a greater number of channels. The API displays limited temporal diversification: the top-20 results for region-language queries performed at different times are mostly the same. Fig 1 shows the fraction of shared videos between top-20 videos retrieved in queries repeated after one and two weeks (queries with less than 20 results not considered). Each data point represents a query for a region/language pair (e.g. Hindi in India, English in India), with the distribution ranging from 0 (no common results) to 1 (all same results). The median percentage of common top-20 videos is high, respectively 85% and 75% after one and two weeks. Moreover, 75% of the queries returned at least 65% and 60% common results after one and two weeks. Since we got 400 results (our self-imposed limit) for almost all searches, we can rule out the possibility that there was no alternative supply.

**Fig 2:**
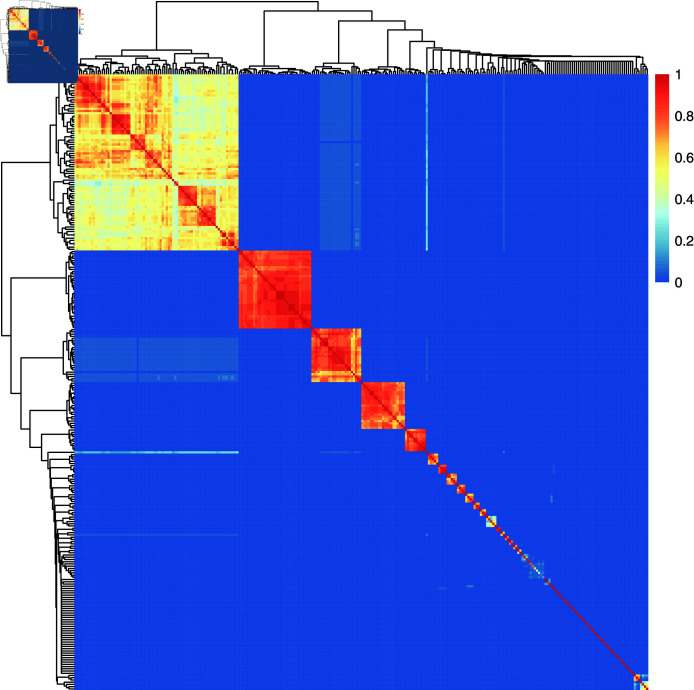
Platform’s geographical diversification. Value 1 corresponds to the case where results obtained from a pair of country-language queries are identical. Value 0 corresponds to query results with no common videos.

Meanwhile, the conditions of visibility for local actors are constrained. The API presents limited regional diversification. Searches in the same language return similar results for different regions and continents, even when the language of the video itself is not in that language. Searches in neighbouring regions with different languages yield significantly different results. We tested on multiple collections, but here present results from a single data collection. Fig 2 shows the overlap, i.e. fraction of shared videos, in top-20 results for all pairs of country-language queries. Each cell shows a pair of queries, and the colour indicates overlap, from no overlap (blue) to complete overlap (red). The clusters correspond to queries with the same language. The five largest are English (79 countries), French (35), Arabic (24), Spanish (21), and Portuguese (10). Similar patterns remain when considering fewer and more results. Importantly, higher ranked results show the least diversification (see supplementary material: diversification, language and country). These language clusters matter for the visibility prospects of local channels. As the results in the previous section showed, channels from particular regions dominate the global language clusters.

**Fig 3:**
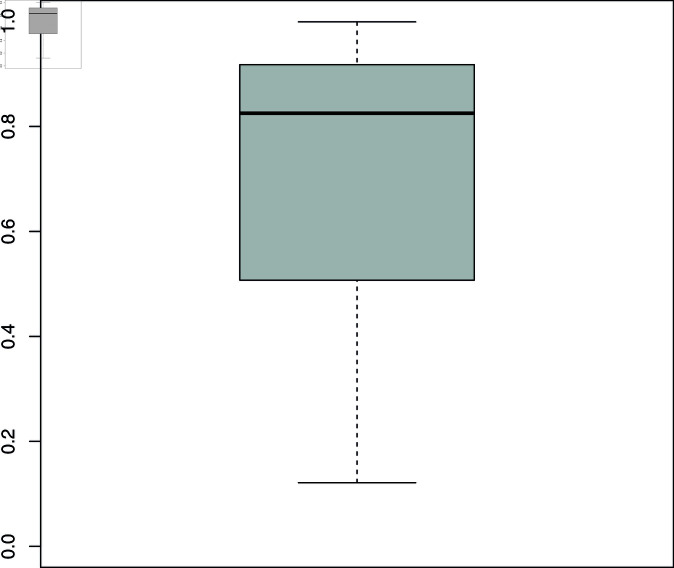
Users’ engagement diversification. Value 0 indicates no diversification, that is, all channel types returned by queries in a given language obtain the same attention expressed in terms of views. Value 1 indicates that one channel type receives all the views for a given language. The plot shows the distribution of engagement diversification over languages.

We next consider the contribution from other sides. While the platform certainly contributes to keeping the rankings constant, it is also possible that audience preferences contribute to sluggish ranking dynamics by persistently favouring particular videos and channels. Given the strength of the language pattern, we continue to organise these results according to language clusters. As Fig 3 shows, there is a clear concentration of preferences for some channel types over others across most language clusters (although not always for the same types). Each value in the boxplot corresponds to the distribution of audience views across channel types in a language (for those with at least five videos in the results). The distribution runs from 0 (equal attention) to 1 (one type receives all attention). This picture holds when considering comments and likes.

**Fig 4:**
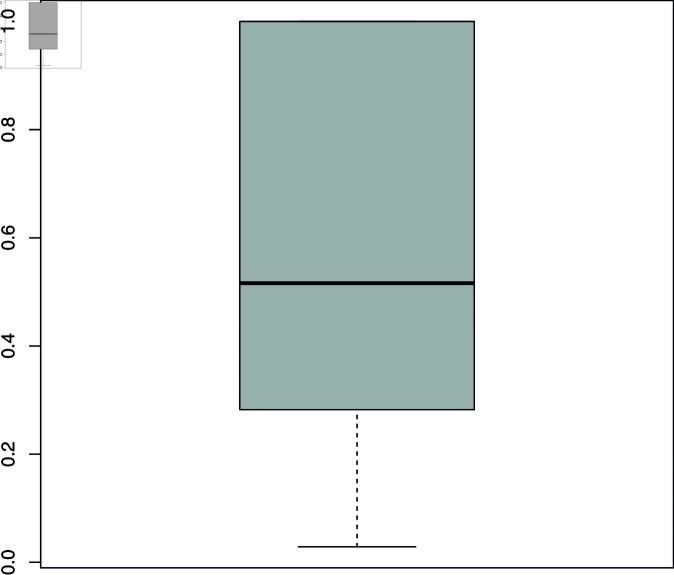
Channels’ content diversification. Value 0 corresponds to the case where all channel types for a given language contain exactly the same fraction of videos with arctic themes. Value 1 corresponds to the case where only one channel type contains videos with arctic themes. The plot shows the distribution of content diversification over languages.

The previous section already established that fresh actors beyond the conventional climate communicators do appear in top rankings, but that the sheer presence of fresh channels does not in itself imply a break with conventional content. Here, probing the channel side confirms that some channel types do tend to include arctic themes more than others. However, this is not predictive: it still varies with the context, even in the case of the professional media channels. Moreover, in many cases, several different channel types together manifest the presence of the arctic repertoire at the top. Fig 4 shows the distribution of channel types with arctic content (with at least 5 top-ranked videos) in top results across contexts. Each value corresponds to a language cluster and distributes from 0 (if all channel types show the same fraction of videos with arctic themes) to 1 (if only one channel type shows them). The median is low, at .52, tending towards distributed contribution. Given the small number of observations, these results at best demonstrate a random variability: at a global level some channel types contribute more than others to the prominence of arctic themes, but the respective sets of top-ranked results are too small to draw conclusions.

Finally, intriguingly, exploratory analysis suggests that the presence of arctic themes seems to intensify at the top. We extended beyond the original research design outlined earlier to analyse top and extended rankings using the Resnet50 pre-trained classifier from the pytorch Python package. The results indicate that polar bear prevalence roughly doubles in top 50 results as compared to top 400 results, across differently profiled contexts such as US/English, France/French, Germany/German, and Portugal/Portuguese. This analysis can only be suggestive, since only a selection of videos was classified and our tests show that the pre-trained classifier scored low in recall for the polar bear theme. Nevertheless, it does raise the tantalising possibility that this classic visual climate repertoire correlates with video rank prominence (see supplementary material: top-ranked results, trends in arctic content).

## 6 Discussion

This study addresses the role of digital intermediaries in online climate discourse in light of the green ghetto. To investigate the expectation that a delimited circle of speakers, stories and styles would become less predominant, it is not enough to consider what climate communicators post. We contend that the role of digital intermediaries requires dedicated attention, and argue for adopting a comparative sensibility. To this end, we analysed how a major global visual platform, YouTube Search, articulates climate change to audiences across the world. The findings invite three key reflections about the role of YouTube as global digital intermediary in online climate communication in general, and the persistence of a green ghetto in particular.

First, the study confirms that global YouTube Search does not dramatically dissolve a (Global North) green ghetto. To a large extent, the top results sustain the status quo, albeit with some modification. Keywords associated with alternative climate discourses would likely yield different patterns in aggregate, and more varied patterns across contexts. Still, these results bear out at global scale earlier findings about climate communication on English-language YouTube. In this mainstream rendition, it is clear that visual climate repertoires from the mass media era are perpetuated online.

The findings underscore the stickiness of the arctic distancing frame as established visual shorthand for climate issues [32,81]. Both old and new climate communicators contribute to its persistence. Climate communication ‘usual suspects’ such as the news media are still prominent in top results, and continue to perpetuate the arctic distancing frame. Fresh actors in the form of user-led and popular science channels widen the circle, but also sustain the distancing theme. We note that the persistence may at times be less about what is actually depicted and more about other functions. For example, the use of these visual themes may be more about pursuing engaging motifs such as animals to anticipate algorithmic visibility or about borrowing legacy footage and frames modelled on professional media as authoritative actors [79].

Second, YouTube’s platform-side language clusters (as observed through the API) create uneven conditions of visibility for relevance in the form of local channels and content. Top results in the global language clusters consistently present audiences with channels from the Global North (with Portuguese as a key exception). They focus geographically disparate audiences — be they in Canada, Cameroon, and Haiti, or in Angola, Mozambique and Portugal — on renditions of the issue primarily sourced in one place, such as, respectively, France or Brazil.

What audiences make of this is less clear. This study cannot discern the resonance, in part because the engagement metrics data for each video is only given in its global form. It is possible that high engagement in one audience obscures the lack of it in another, or that geographically remote channels are still experienced as relevant due to shared historical or media hub connections. Another possibility is that no matter the starting point, the arrangements inadvertently create new publics for old visual repertoires, as they are reinterpreted and incorporated into local repertoires, possibly with critical overtures [82]. More contextualising approaches that can consider for example full video sequences and comments are needed to sort such questions out.

Finally, it is crucial to address how global platforms operate in different contexts. The findings underscore the need to attend to differences not only across platforms, but also within them. Studies of online climate communication typically build on the US context and/or English-language data, and the external validity is often assumed. Yet, this can be misleading. In this case, there were clear continuities between global YouTube Search contexts, but also significant exceptions. For example, US/English YouTube was close to the norm and to other contexts such as India/English. However, it was far from the profile of other language groups with sizeable audiences, such as Hindi and Portuguese. Being too quick to assume one size fits all risks overlooking local narratives [55], and ascribing local circumstances too broadly. In order to understand the role of digital intermediaries in online climate communication, we need to think beyond the notion that global digital intermediaries have only one face.

## Supporting information

SI1 Supplementary material. This document provides coding schemes for channels and visual themes, the definition of the measures used in this article, and additional summaries of top-ranked API query results.
(PDF)
