# Supplementary material for: Visual digital intermediaries and global climate communication: Is climate change still a distant problem on YouTube?
Source: PLoS One. 2025 Apr 8;20(4):e0318338. doi: 10.1371/journal.pone.0318338 (PMC11978093; doi:10.1371/journal.pone.0318338)
Supplement: SI1 — This document provides coding schemes for channels and visual themes, the definition of the measures used in this article, and additional summaries of top-ranked API query results. (PDF) [file pone.0318338.s001.pdf]

Visual digital intermediaries and global climate communication: is climate change still a distant problem on YouTube?

Supplementary material

Alexandra Segerberg* and Matteo Magnani†

Coding schemes

Channel categories

We coded the channels with one or more video(s) in the top-20 results by category to analyse the composition and concentration of channel actor types. The data presented in the main article contains 1720 unique videos and 1299 unique channels. However, to check the stability of our results we performed two additional data collections with the same search parameters, leading to a larger dataset containing 2266 videos and 1631 channels, of which all were coded.

As noted in the main text, the codebook draws on and develops coding schemes used in prior research to identify key climate communicator types, in order to capture not only those that are highly visible but also those that may be active but not prominent. Table S1 shows the 17 categories, which include traditionally prominent actors (e.g., media, science, and civil society organisations), well-established actors that have not previously been prominent in public debate or research (e.g., businesses, politicians and political parties, religious organisations), and newly emerged actors (e.g., user-led channels, popular science groups, online education initiatives). Coders assessed the name, channel description and links in channel description. Channels with neither description nor link were coded as No description. The coding was based on explicit information about the channel, not on assessments of genre, content or amateur/professional status.

The coding framework was developed and refined in several iterations. With the first rendition of the codebook, two researchers coded a random selection of 10% of the channels in the larger dataset of 1631 channels. After assessing inter-coder reliability, we refined the codebook by merging closely related categories, clarifying instructions, and undertaking further training. The final codebook is presented in Table 1. The Cohen's kappa inter-coder reliability was .81, based on the annotations reported in Table 2. As can be seen in the Table, there was some disagreement on the category Popular science group, a category that became important in the analysis. This disagreement was straightforward to resolve, as it had to do with miscommunication regarding the focus on non-

*Dept. of Government, Uppsala University, Sweden
†InfoLab, Dept. of Information Technology, Uppsala University, Sweden

institutional groups dedicated to popular science communication (as opposed to individuals, public intellectuals, or school classes).

Visual themes

We coded videos by content in terms of visual arctic themes, extracting representative frames from each video as described in the main text and looking for the depiction of polar bears, icebergs, and ice cliffs in water. The codebook is provided in Table 3. This codebook has a different character than the one used for coding channel categories (Table 1), in that the coding instructions apply in part to qualitative manual coding and in part to computational image classifiers, in this case, artificial neural networks. Note also that we used distinct coding processes for the visual themes of polar bears and arctic sea landscape, since they presented different coding challenges.

Polar bears

The polar bear analysis was straightforward. To identify polar bears in the data, we used an ensemble of neural networks designed to identify them (i.e., with output nodes associated specifically to polar bear pictures.) In addition to two pre-trained networks (Google Vision API and Resnet50 from the pytorch Python package), we trained two custom networks, then combined first the two custom networks and then all of them into a single classifier. Training an image classifier requires images with and without polar bears, so that the training algorithm can find weights for the links in the neural network that discriminate between the two classes. To train our custom networks, for the positive classes, we used 452 photos of natural polar bears and 204 drawings of polar bears obtained through Google Search. For the negative classes, we extracted random YouTube images from the data used to perform this study, manually checked not to contain polar bears.

Since identifying polar bears manually was uncontroversial — there was full agreement on whether frames contained polar bears — we concentrated our validity assessment to the classifier. For classifiers, validity has two aspects: precision (the fraction of identified frames that do contain polar bears) and recall (the fraction of polar bears in the data that are correctly identified). We aimed to train high-recall networks. Under the assumption that a small set of frames is identified as containing polar bears, precision is not particularly important — manual inspection of this subset removes all false positive (i.e., no error is associated to a precision lower than 1). Under the assumption that the dataset is too large for manual analysis, recall cannot be computed, and positive frames not identified by the network may affect the validity of the analysis. In this study both assumptions hold, making training for high recall more urgent.

To assess validity, we tested our networks on different datasets. This process also increased our understanding of what the network identifies as 'polarbearness'. Table 4 presents the validity analysis for the polar bear networks based on four datasets. Control does not contain any polar bears, so high-precision networks should positively label no or only a few pictures. Our (combined) custom-trained network labels 4% of the pictures, and is thus less precise than the others. This is expected since the network was trained to increase recall; false positives are manually removed and do not result in errors. The custom-trained network correctly identifies more pictures for both datasets from Wikimedia commons. For drawings, only a 63% recall is achieved, which is expected due to the many ways in which a polar bear can be drawn. The pre-trained networks show a significantly lower recall in both cases. Notably, however, the pre-trained network Resnet50 labels more pictures from the Greenpeace data (which contains activists dressed as polar bears) than the custom-trained one, which was trained on natural polar bears.

Category	Explanation	Example
(Inter)govern. organisation	Public agencies, governmental and intergovernmental organisations. Included: state meteorological institutes, and affiliated campaign channels.	EU Climate Action; Meteo France; United Nations; ONU Brasil; Swedish Environmental Protection Agency.
Business	Business associations and large companies. Not included: self-employed businesses or semi-professional youtubers, companies that are covered by other categories (e.g., online educations companies, which are classed as online education).	Siemens; IKEA Iceland; Enel Chile.
Civil society	Non-governmental organisations, explicit activist groups and collectives, foundations, think-tanks, including student radio and dedicated collective interest sites.	Greenpeace Polska; WWF Danmark; Oxfam in Laos; Ellen MacArthur Foundation.
Digital media producer	Production company that produces online media content for others.	Spring; Genomedia.
Extreme weather channel	Channels explicitly, exclusively, or predominantly devoted to extreme weather content.	Dangerous Planet.
Music	Music producers (e.g. record label), or musician groups (e.g. band, orchestra).	See-Saw Production Fiji; Jazz i trekanten.
Online education	Organisation, company, or initiative focused on online school education. Includes: for profit or not, legacy or digitally native. Does not include: individual teachers, school classes with science projects or government initiatives.	Curso enem gratuito; HUB Scuola; Knowledge Hub SL.
Politician, party & parliament	Individual politicians in institutional politics, parties, parliament (e.g., live-streaming debates).	Knesset Channel; Rep. Dan Crenshaw; Sósíalistaflokkurinn.
Popular science group	Groups dedicated to popular science communication, including news, animation or entertainment. Includes: YouTuber groups that may also now be broadcast on professional media. Does not include: personal or individual projects; professional media organisations; school classes.	Kurzgesagt; ASAPScience; Nauka; to Lubię.
Professional media organisation	Legacy, digitally native, and public service media organisations that produce professional news, journalism, and entertainment. Includes digitally-native styled platforms and content produced by these organisations.	BBC News; Voice of America Deewa; France 24 Arabic; DW Deutsch; 60 Minutes Australia; CNBC Indonesia
Religious organisation	Religious institutions and organisations (e.g., churches, synagogues, organised missions, group-run dedicated channels). Does not include: personal or one-person religious channels.	Salisbury SDA Church; Midway Samoan AG Church; Centenary Methodist Church Fiji.
Science organisation	Universities, museums, research networks, institutional science communication initiatives.	Cambridge University; Vilnius University; Klimawandel (Ludwig-Maximilian University initiative).
Talk platform	Online lecture platform.	Ted Talks; TedX.
User-led channel	Includes: Personal channels, individual youtubers, school classes, public intellectuals.	Date un Vlog; The Origins Podcast.
No description	No description, no link.	—
Channel discontinued	Channel discontinued.	—
Other	Other, insufficient information.	—

Table 1: Coding scheme, categories

	Coder 1																
Coder 2	IGO	B	CD	CS	DMP	EWC	M	ND	OEC	Polit.	PSG	PMO	RO	SO	TP	ULC	Tot.
(Inter)govern. org.	10																10
Business		2										1					3
Channel discontinued			4														4
Civil society		1		4				1									6
Digital media producer					2												2
Extreme weather ch.																1	1
Music							2										2
No description				1				23								2	26
Online edu. company									2		1						3
Politician, party, parl.										1							1
Popular science groups									1		4				1	3	9
Professional media org.											3	38					41
Religious organisation													1				1
Science organisation	1			2										5			8
Talk platform															2		2
User-led channel		1					1		1		3	1				35	42
Tot.	11	4	4	7	2	0	3	24	4	1	11	40	1	5	3	41	161

Table 2: Inter-coder agreement by category on 161 channels (10% of the channels)

Category	Explanation	Examples	Classifier
Polar bear	Presence of a polar bear, including cartoons and drawings.	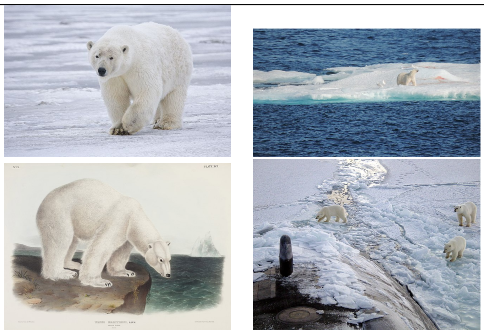	Google vision API with label polarbear.<br>Resnet50 with label ice_bear.<br>Custom-trained networks for: wild polar bears, polar bear drawings.<br>Manual filtering.
Ice cliff	Vertical ice cliff drop of significant height in water. The water can contain ice. The water must be in the picture. Cliff is in a prominent position in the picture; part of it in the center or at least 1/8th of the picture. A cracked ice cap is not accepted.	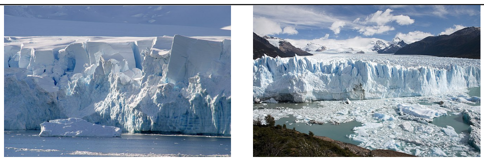	Google vision API with labels: Glacial landform, Polar ice cap, Ice cap, Arctic ocean, Iceberg.<br>Manual filtering.
Iceberg	Iceberg, of which full extent visible inside the picture. Must be protruding (flat sheets of ice in water are not accepted). Iceberg is in a prominent position in the picture: part of it in the center or at least 1/8th of the picture. It is isolated (no archipelago; only one or a few large icebergs). It must be free-floating: not connected to shore.	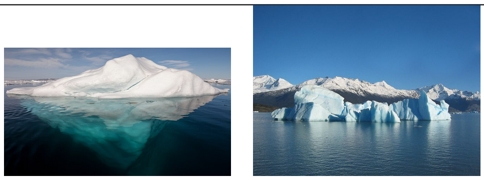	Google vision API with labels: Glacial landform, Polar ice cap, Ice cap, Arctic ocean, Iceberg.<br>Manual filtering.
Reject	Anything not conforming to our definition of ice cliff or iceberg. Anything showing visible human presence (but acceptable if people showing pictures, eg. studio presenters).	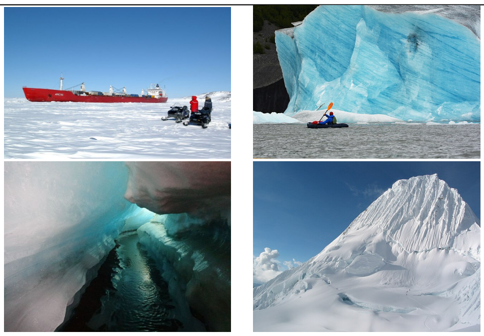	Google vision API with labels: Glacial landform, Polar ice cap, Ice cap, Arctic ocean, Iceberg.<br>Manual filtering.

Table 3: Coding scheme, visual themes

The combination of networks identified 232 positive frames. Table 5 shows the contribution of different neural networks to detecting the identified frames with polar bears. The custom-trained network alone identifies most of the results (91%), but all networks correctly identified polar bears that the others missed. Some additional results were found in the analysis for arctic sea landscapes and added to the analysis. Note that a false negative does not necessarily generate an error in the analysis, since a video is labelled as positive if any of its frames are labelled as positive.

Icebergs and ice cliffs in water

The arctic landscape analysis posed a different set of challenges, since it was unexpectedly ambiguous what should be considered as part of this theme. We developed a coding scheme that aims to capture a conservative rendition of the deserted arctic sea landscape that has been typical of 'distancing' mainstream climate communication in the past decades. By *conservative*, we mean that we restricted focus to the classic type of images of icebergs and ice cliffs in the water, and not other icy landscapes that in principle could also be considered distancing but are not as iconic, such as mountain-top glaciers. However, we did not require that the location was confirmed as arctic.

We developed and refined the codebook in several iterations. To identify icebergs and ice cliffs, we first extracted images labelled as *Glacial landform, Polar ice cap, Ice cap, Arctic ocean*, or *Iceberg* by the Google vision API, then manually coded the selected images. We removed all frames clearly not relevant for the analysis (e.g., those not containing any ice), then clustered the remaining frames based on features that we considered important for making a decision (e.g., close-ups of ice with no landscape, alps, pictures of people, etc). Sample images were independently classified by two coders and then discussed with respect to their thematic relevance to our conservative definition and the ability to code them reliably. The final scheme is presented in Table 3. The final coding identified 575 positive frames.

In contrast to the polar bear analysis, we assessed the validity for both the manual qualitative and network parts of this coding scheme. For the former, we tested inter-coder reliability between two human coders on a sample of 187 images, obtaining a Krippendorff's alpha equal to .84. For the latter, the neural network applied on 50 positive examples from Wikimedia commons showed a recall of 92%.

Measures

The values reported in the tables for prevalence, amplification, composition, content, and concentration are based on the following definitions.

Definition 1 (Prevalence)**.** *Given N search results, with N_c results corresponding to a video posted by a channel in category c, the prevalence of category c is $\frac{N_c}{N}$.*

Example 1 (Prevalence)**.** *Given the following search results:*

Country	*Video*	*Channel*	*Category*	*Icebergs*
Country 1	*Vid1*	*Ch1*	*CatA*	*yes*
Country 1	*Vid2*	*Ch1*	*CatA*	*no*
Country 1	*Vid3*	*Ch2*	*CatA*	*no*
Country 2	*Vid4*	*Ch3*	*CatB*	*no*
Country 2	*Vid5*	*Ch3*	*CatB*	*no*
Country 2	*Vid1*	*Ch1*	*CatA*	*yes*

The prevalence of category CatA is $\frac{4}{6}$.

Definition 2 (Amplification). *Given* N *search results containing* V *distinct videos, with* N_c *results corresponding to a video posted by a channel in category* c*, and* V_c *distinct videos posted by channels in category* c*, the amplification of category* c *is* $\frac{V N_c}{V_c N} - 1$.

Example 2 (Amplification). *In the example above, the amplification of category CatA is* $\frac{5*4}{3*6} - 1 = 0.1$*, that is, a positive amplification, indicating that videos by channels in category CatA are more likely to appear in search results across different countries than the number of distinct videos would suggest.*

Definition 3 (Composition). *The composition of a set of search results is the prevalence of each category represented in the set.*

Example 3 (Composition). *In the example above, composition is: CatA:* 67%*, CatB:* 33%*.*

Concentration is a measure of inequality of distribution. Low concentration of a search result is associated to the presence of many categories, each with a similar number of search results. High concentration indicates that most search results belong to one or few categories.

We use two measures of concentration. The max-based definition only looks at the most represented category.

Definition 4 (Concentration: classification error). *Given* N *search results, with* N_c *results corresponding to a video posted by a channel in category* $c \in C$*, the concentration of the search results is defined as:* $\max_{c \in C} \frac{N_c}{N}$.

This measure is easy to understand, and works well if one category covers a large fraction of the results. However, max-based concentration does not differentiate between a case where two categories cover 50% of the results each and a case where one category covers 50% of the results and the remaining results are equally distributed among several categories. For this reason, we also use a definition based on entropy, which would identify the former case as more concentrated.

Definition 5 (Concentration: entropy). *Given* N *search results, with* N_c *results corresponding to a video posted by a channel in category* $c \in C$*, entropy is defined as:* $E = \sum_{c \in C} -\frac{N_c}{N} \log_2 \frac{N_c}{N}$. *Concentration is defined as* $1 - E/\max(E)$*, where* $\max(E)$ *is the maximum possible entropy obtainable with 17 categories.*

For high concentrations, the two definitions show similar results: 7 of the 10 most concentrated languages are common. However, there can be significant changes of ranking. The most relevant example is German, which is the 4th most concentrated language using the max-based definition and the 18th most concentrated using entropy (still in the top quartile).

Example 4 (Concentration)**.** *In the example above, the concentration is* $\frac{4}{6} = .67$ *if computed with the max definition.*

Definition 6 (Content)**.** *For each visual theme (PolarBear, Iceberg and Cliff) we define content as the fraction of results where the video includes at least one frame belonging to the theme.*

Example 5 (Content)**.** *In the example above, content for Icebergs is* 50% *for category CatA and* 0% *for category CatB.*

Diversification

Time

We tested the extent to which repeating the same query (same language, region, and time window) at different times produces similar results. We do this for two reasons. High similarity indicates that YouTube consistently promotes (and thus amplifies) the same few top results. A higher similarity is also associated to a higher generalisability of our results (i.e. that they are not an artefact of this specific data collection).

Figure S1 shows the similarity of top-k results, measured as the fraction of common videos, for a single query (region: Great Britain; language: English; time window: until December 31, 2020) repeated at different times (on the same day on February 24, after two days on February 26, after three months on May 21, 2021), for k = 5, 20, 50, 100. In this experiment, the immediate repetition of the same query produced the same top results. Three of the top-5 (60%) and more than 50% of the top-20 results are still the same after three months. Even if we constrain the queries to return videos from the same time window, the videos' engagement metrics (used in the ranking algorithm) may change in time. There may be some diversification, but we cannot conclude that the observed differences are due to YouTube actively trying to avoid promoting only the same few top results.

Language and country

In the main text we present the overlap in top-k results varying country and language, for k = 20, showing the emergence of language-based clusters. We checked if this pattern was visible also for other values of k. Figures S2, S3, and S4 show the same pattern when we consider less and more top results, respectively 5, 50, and 100.

The reason to look at deeper search results (higher k's) is that the presence of language-based clusters, which include geographically distributed country regions that have largely overlapping results, suggests that search results in some regions are associated with non-local content. We considered the possibility that a few top-ranked globally popular videos are shown in all regions with the same language but are followed by distinct search results for each region. However, we did not observe support for this possible pattern.

The plots suggest the existence of sub-clusters inside the major languages. However, this is likely an artefact of the data collection process. We believe the sub-squares with higher values of overlap inside the main squares to be the result of queries performed on different days. Because of quota limitations, a single world-wide data collection required up to one week. As can be seen in Figure S1, results can vary when the same search is executed on different days.

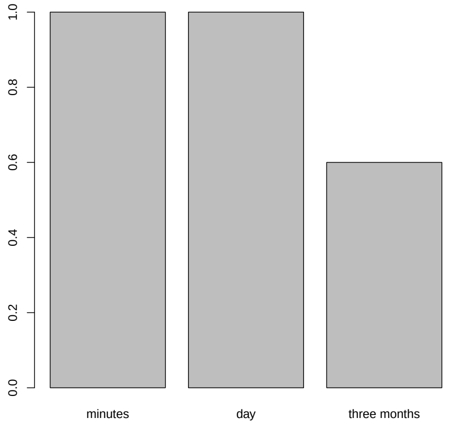

(a) k = 5

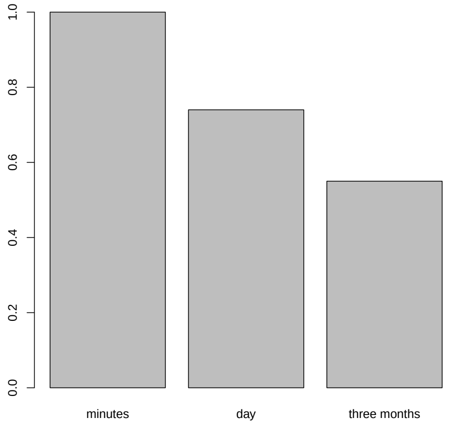

(b) k = 20

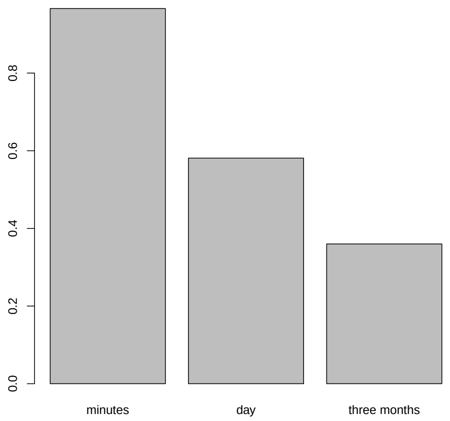

(c) k = 50

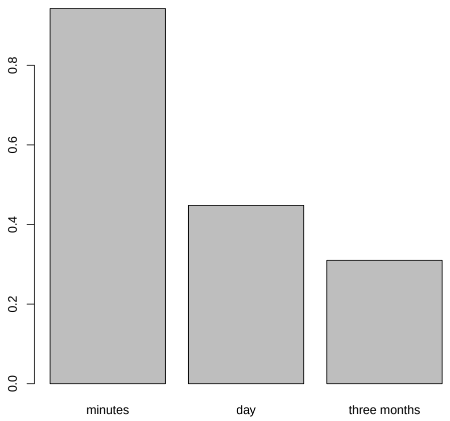

(d) k = 100

Figure S1: Fraction of common top-k videos when a query is repeated a few minutes later, two days later, and three months later (Country: Great Britain, Language: English)

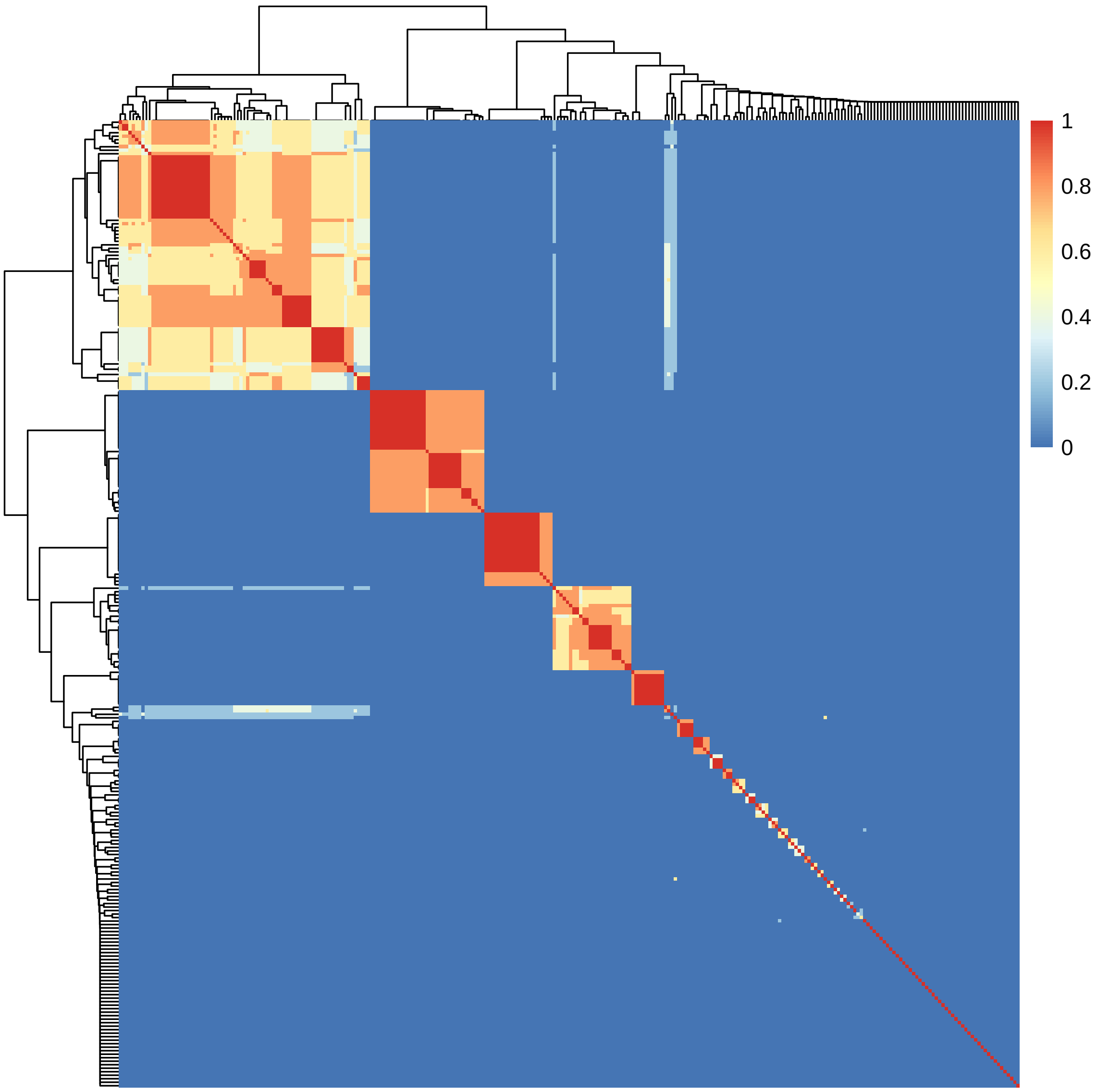

Figure S2: Overlapping of top-5 videos varying Country and Language. Color figure.

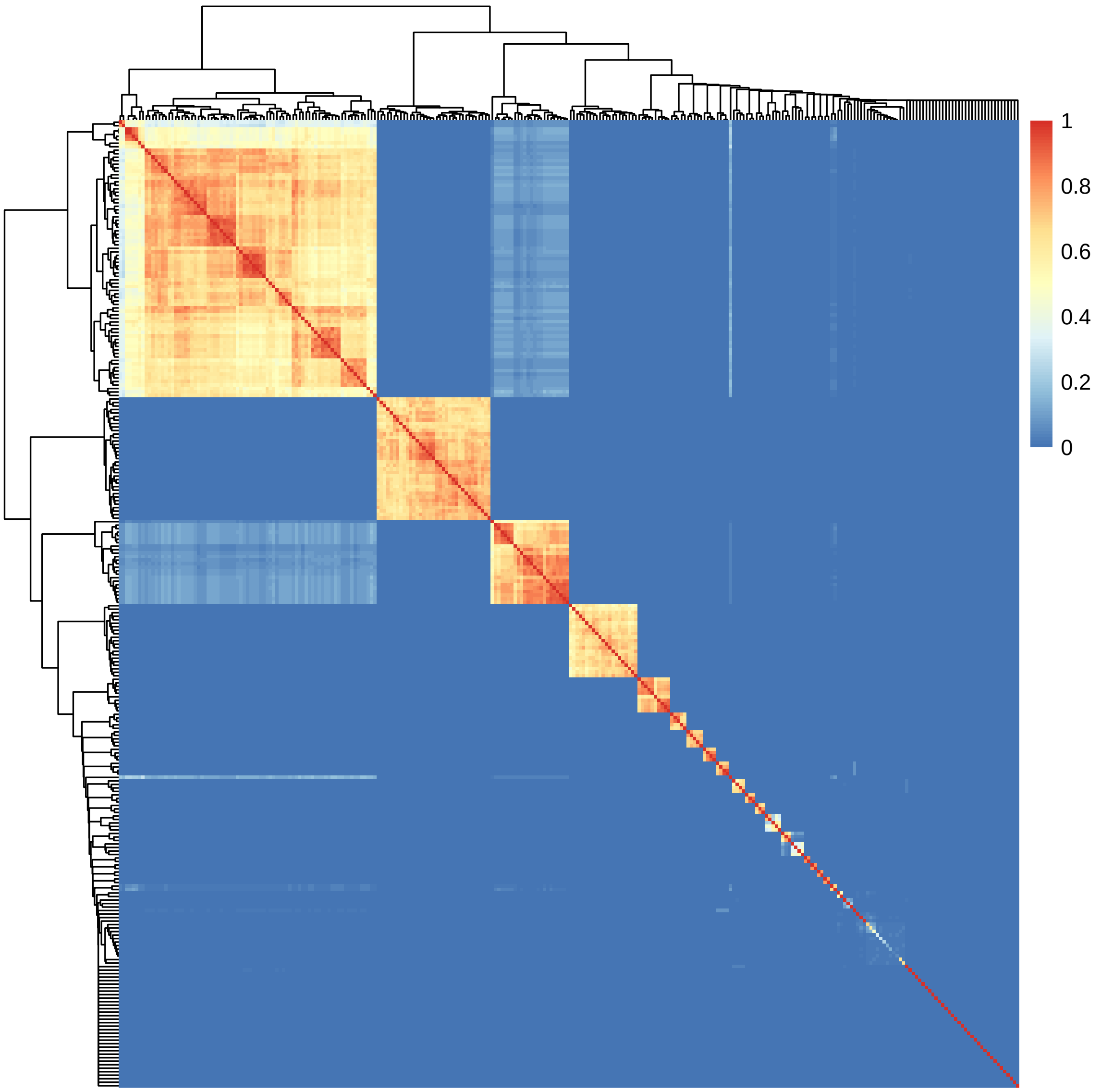

Figure S3: Overlapping of top-50 videos varying Country and Language. Color figure.

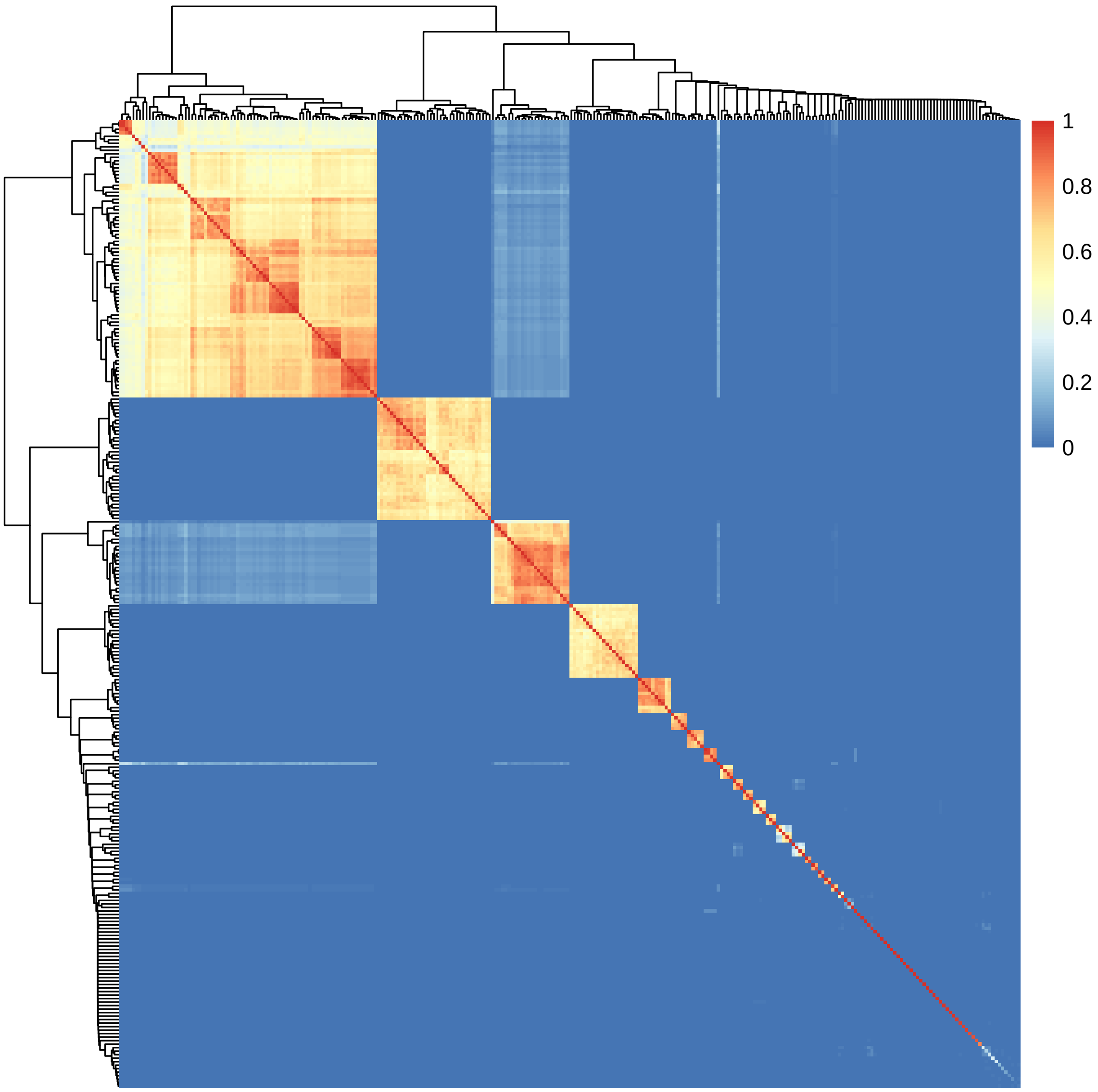

Figure S4: Overlapping of top-100 videos varying Country and Language. Color figure.

Audience

Figures S5 shows the distribution of audience likes and comments across channel types in a language, for those with at least 5 videos in the results (0 = equal attention, 1 = only one channel type receives all the attention).

Top-ranked results

Channel concentration by language

For the top-10 most concentrated languages in descending order, using entropy as a measure of concentration, the most represented category for each language is shown in Table 6.

Channel location by language

Table 7 shows in which countries channels with videos in top search results in different languages are based. We show three different cases: one with a strong dominance of a few countries, but also a long list of other countries represented in some local searches (English), one with most channels associated to the same country (Portuguese), and one with a less concentrated distribution of countries (Arabic). Tables 8 and 9 show the channels appearing in the top-5 (English) and top-20 (Arabic, French) search results.

	#pictures	Google Vision (polarbear)	Resnet50	Custom
Control	179	0	1	7
Commons	91	80	85	90
Commons draw	8	3	4	5
Greenpeace	52	13	26	19

Table 4: Classifier validity analysis. Number of pictures labelled as containing polar bears when our networks are applied to four datasets. Control contains 179 frames from our data not containing polar bears. Commons and Commons draw contain depictions of polar bears from Wikimedia commons. Greenpeace contains photos from the website of Greenpeace, mostly showing people dressed up as polar bears.

	Google Vision (polarbear)	Google Vision (ice)	Resnet50	Custom
Google Vision (polarbear)	**119**			
Google Vision (ice)	81	**132**		
Resnet50	102	97	**147**	
Custom	118	111	147	**210**

Table 5: Contribution of different neural networks towards the 232 identified video frames. The diagonal indicates the number of frames identified by each network. Entry (A,B) indicates the number of frames identified by both A and B.

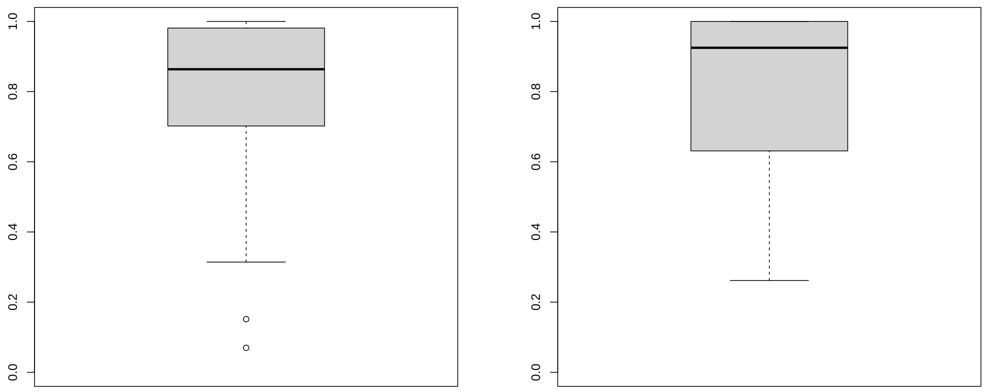

(a) Likes (b) Comments

Figure S5: Users' engagement diversification.

Lang.	Conc.	Top category
tk	.82	User-led channel
sq	.80	Professional media organizations
tg	.69	User-led channel
so	.68	Professional media organizations
am	.67	Professional media organizations
zu	.61	User-led channel
bs	.61	Professional media organizations
ar	.61	Professional media organizations
mg	.60	No description
bg	.60	Professional media organizations

Table 6: Most concentrated languages with their most represented channel category. Concentration calculated using the entropy-based approach.

English		Portuguese		Arabic	
Country	#Ch	Country	#Ch	Country	#Ch
US	24	BR	24	AE	4
GB	10	ES	1	DE	3
DE	5	N/A	6	JO	2
IN	4			SA	2
CA	4			US	1
PH	3			TR	1
AU	3			LB	1
UA	1			GB	1
SI	1			FR	1
SG	1			EG	1
QA	1			BH	1
PK	1			N/A	8
BD	1				
AE	1				
N/A	13				

Table 7: Number of channels appearing in top twenty results of all searches in English, Portuguese, and Arabic, grouped by country in the channel metadata. N/A: Not Available.

Channel	Country
ABC News	US
CNBC Television	US
CrashCourse	US
National Geographic	US
Our Changing Climate	US
Second Thought	US
TDC	US
BBC	GB
Junaid Akram	GB
Sky News	GB
9 News Australia	AU
ABC News In-depth	AU
Dhruv Rathee	DE
Kurzgesagt – In a Nutshell	DE
Global News	CA
GMA News	PH
Mushtaq Ahmad	PK
Al Jazeera English	QA
Climate change	UA
CloverTV	N/A
Matt Miltonberger	N/A
The New York Times	N/A
The not so boring Man	N/A

Table 8: Channels appearing in top five results of all searches in English, by country in the channel metadata. N/A: Not Available.

Arabic		French	
Channel	Country	Channel	Country
Al Aan TV تلفزيون الآن	AE	1 jour 1 question	FR
AlHadath الحدث	AE	28 minutes - ARTE	FR
Genomedia	AE	ARTE Découverte	FR
سكاي نيوز عربية	AE	Agence de l'eau Adour-Garonne	FR
SCE BH	BH	Cistude Nature	FR
DW Documentary وثائقية دي دبليو	DE	France Culture	FR
DW السلطة الخامسة	DE	Futura	FR
Kurzgesagt – In a Nutshell	DE	Greenletter	FR
تعلم - TAALLAM	EG	JEAN-LUC MÉLENCHON	FR
فرانس 24 / FRANCE 24 Arabic	FR	L'Obs	FR
BBC News عربي	GB	Le Monde	FR
AlMamlaka TV - قناة المملكة	JO	Le monde en cartes	FR
اعرف اكثر	JO	LeTopDix	FR
هنا لبنان - This is lebanon	LB	Les Décrypteurs	FR
أحداث وحقائق	SA	METEO FRANCE	FR
فريق تاء التطوعي	SA	Nous Sommes Pour	FR
TRT عربي	TR	Osons Causer	FR
قناة الحرة - Alhurra	US	T C'EST ÇA LÀ MÊME	FR
27Celsius	N/A	franceinfo	FR
AlArabiya العربية	N/A	la chaîne humaine	FR
Forbes Middle East	N/A	Jonathan Dumas	BE
GIZ	N/A	World Bad News.	BE
euronews (عـربي)	N/A	36.9	CH
الصحفي محمد الغرباوي	N/A	Université de Genève (UNIGE)	CH
اللسان الانجليزي	N/A	Les Panafricaines	MA
فوائد اسلامية	N/A	AFP	N/A
		Data Gueule	N/A
		Epanouissement Authentique	N/A
		GoodPlanet	N/A
		Jean-Marc Jancovici	N/A
		PNUD Madagascar	N/A
		malignedemire95	N/A

Table 9: Channels appearing in top twenty results of all searches in Arabic and Franch, ordered by country in the channel metadata. N/A: Not Available.

Table 10: Trends in arctic content

classes	top-50	top-100	top-150	top-200	top-250	top-300	top-350	top-400
US, English								
no PB	36	61	80	100	125	143	157	174
PB	14	20	29	31	35	37	39	41
unclassified	0	19	41	69	90	120	154	185
ratio PB/classified	0.28	0.25	0.27	0.24	0.22	0.21	0.20	0.19
France, French								
no PB	34	61	85	105	115	127	138	147
PB	16	21	22	23	27	28	30	30
unclassified	0	18	43	72	108	145	182	223
ratio PB/classified	0.32	0.26	0.21	0.18	0.19	0.18	0.18	0.17
Germany, German								
no PB	39	61	72	85	94	102	115	127
PB	10	12	15	16	18	18	18	18
unclassified	1	27	63	99	138	180.00	217	255
ratio PB/classified	0.20	0.16	0.17	0.16	0.16	0.15	0.14	0.12
Portugal, Portuguese								
no PB	45	720	920	1080	1240	1380	1530	1650
PB	5	50	50	60	80	90	100	100
unclassified	0	230	530	860	1180	1530	1870	2250
ratio PB/classified	0.10	0.06	0.05	0.05	0.06	0.06	0.06	0.06

Trends in arctic content

Table 10 shows, for four country/language combinations, an estimation of the proportion of videos showing polar bears in the top results, from top-50 to top-400. For the classification we used the Resnet50 pre-trained classifier from the pytorch Python package, with label *icebear* and no manual validation. We selected a random sample of videos to annotate from the top-400 results. For each set of top-k results (k = 50 to 400), we indicate the number of annotated videos (showing how many contained or not frames classified as polar bears), the number of unclassified videos, and the proportion of videos showing polar bears (according to the classifier) among the classified ones.

Credits

All images used in this document are from Wikimedia Commons. Credits go to, in the order they appear: Alan Wilson, Kerry Raymond, John James Audubon, Chief Yeoman Alphonso Braggs, US-Navy (polar bears), AWeith, IlyaHaykinson (icebergs), W. Bulach, Luca Galuzzi (cliffs), Jcmurphy, Paxson Woelber, Superchilum, Brad MeringBaltimore, MD, United States (negative).
